# Research on air quality prediction based on improved long short-term memory network algorithm

**DOI:** 10.7717/peerj-cs.1187

**Published:** 2022-12-20

**Authors:** Wenchao Huang, Yu Cao, Xu Cheng, Zongkai Guo

**Affiliations:** 1 School of Information and Control Engineering, Liaoning Petrochemical University, Fushun, Liaoning, China; 2 School of Economics and Management, Shenyang Agricultural University, Shenyang, Liaoning, China; 3 Liaoning Meteorological Equipment Support Center, Shenyang, Liaoning, China

**Keywords:** Air quality, GRU, LSTM, Bi-LSTM, Attention mechanism, LightGBM

## Abstract

Air quality is changing due to the influence of industry, agriculture, people’s living activities and other factors. Traditional machine learning methods generally do not consider the time series of the data itself and cannot handle long-range dependencies, thus ignoring information relevant to the predicted items and affecting the accuracy of air quality predictions. Therefore, an attention mechanism is introduced based on the long short term memory network model (LSTM), which attenuates unimportant information by controlling the proportion of the weight distribution. Finally, an integrated lightGBM+LSTM-attention model was constructed based on the light gradient boosting machine (lightGBM), and the prediction results were compared with those of 11 models. The experimental results show that the integrated model constructed in this article performs better, with the coefficient of determination (R2) of prediction accuracy reaching 0.969 and the root mean square error (RMSE) improving by 5.09, 4.94, 4.85 and 4.0 respectively compared to other models, verifying the superiority of the model.

## Introduction

Air quality prediction is a popular research field; through the prediction of PM2.5 concentration, one can predict the future development trend of air quality, provide certain support for the implementation of some policies, and have a certain reference value for people to make travel plans. Therefore, improving the accuracy of prediction has always been the goal of many scholars. In the past, scholars have mostly used a single statistical method for air quality predictions. In Li et al. (2017), the Markov chain is used to determine the future development trend of CO and O_3_. Zhang & Huang (2018) established a weighted Markov chain by improving the model, and the accuracy of air quality prediction can reach 85.96%. Wang et al. (2019) established an ARIMA prediction model, and the lowest relative error can reach −4.29%. Machine learning methods can effectively deal with problems with many variables, and many scholars prefer this method. Ren & Xie (2019) used random forest algorithm, and the accuracy of air quality prediction can reach more than 79%. Xu (2021) improved the random forest model through feature selection and weighted random forest, and the prediction accuracy can reach up to 0.83. Neural networks are also widely used to predict air quality. Tang (2018) constructed back propagation neural network (BP) to predict PM2.5 concentration, the relative error is set within 30%, and the accuracy rate is 70.9%. Tan (2021) used Particle Swarm Optimization (PSO) to improve BP neural network, and the prediction accuracy can reach more than 85%. Chen, Tian & Wu (2020) mixed the information gain with a long short-term memory network, and the improved model prediction accuracy reached 0.967. Today, LSTM is also used widely for air quality predictions, and these studies can be found in literature surveys (Liu, Zhang & Qi, 2022; Bai & Shen, 2019; Zhou et al., 2020), the accuracy is up to 0.95. Yu (2020) uses one-dimensional convolutional kernels to extract features, combines LSTM model and genetic algorithm to construct the model, and the MAE is as low as 0.961. In Wang et al. (2020), the cardinality test is used to determine the factors influencing air quality, and then the LSTM is constructed to achieve prediction, and the prediction accuracy can reach 93.7%. Menares et al. (2021) used LSTM and a deep feedforward neural network (DFFNN) to predict the PM2.5 concentration value respectively, and the LSTM gives the best results of 0.87.

The above models improve prediction accuracy to varying degrees, but as the length of the time series increases, information that is more distant from the current information will be lost. This article introduces time series, then reconstructs the relevant data collected from the Beijing Changping and Shunyi weather stations through sliding windows, and builds an attention mechanism-based LSTM model (LSTM-Attention) to solve the problem, then introduces the lightGBM model to construct an integrated LightGBM+LSTM-Attention model. The integrated model can not only solve the problem of easy disappearance of gradients in time series problems, but also assign more weights to factors that have a greater impact on the prediction items. By learning to update the weights of different information (Zhang & Yang, 2021) strengthening key features and weakening unimportant ones. It is important for improving the prediction of PM2.5 concentration.

## Materials and Methods

### Data description

**Figure 1:**
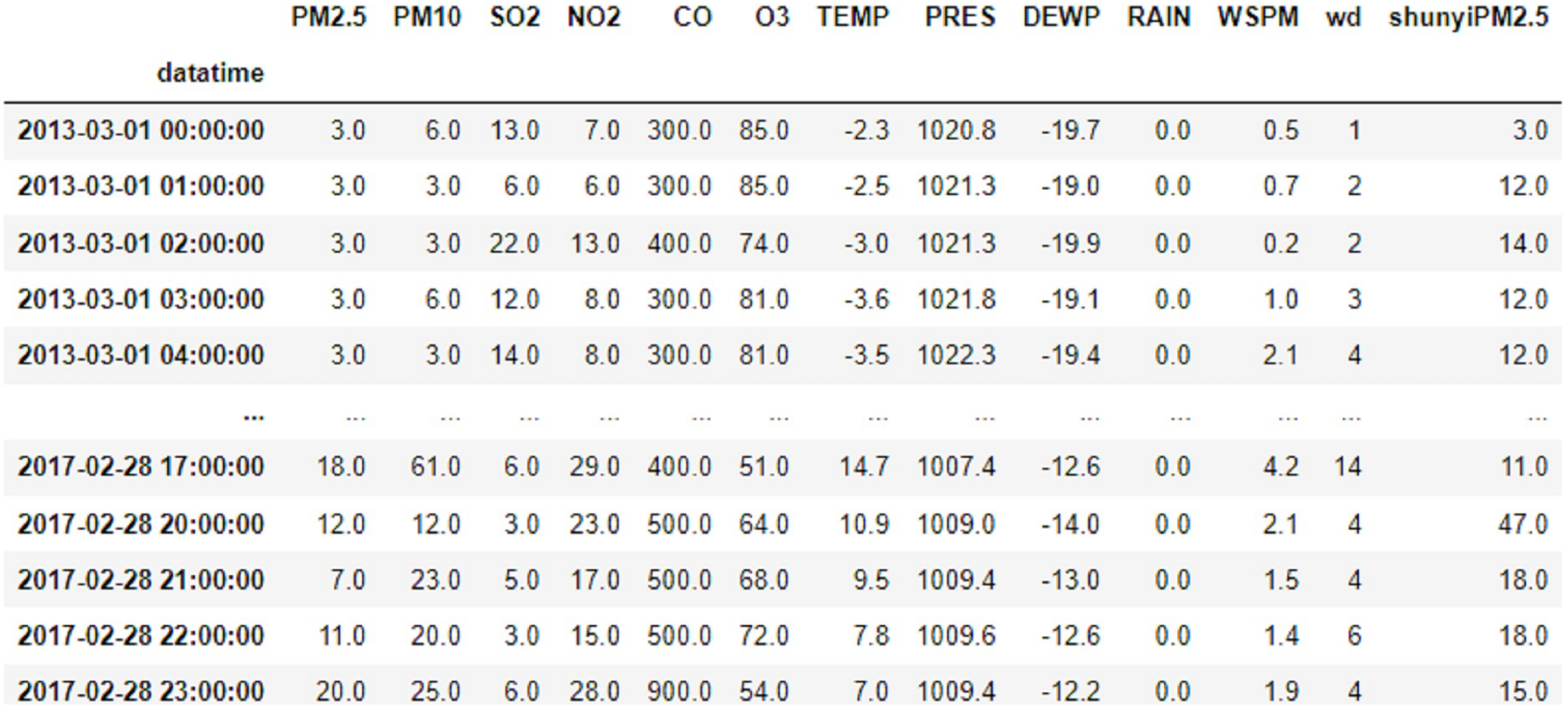
Data example diagram.

This article uses data related to air quality from the meteorological stations in Changping and Shunyi, Beijing, from the UCI machine learning library. The data covers the time span from 00:00 h on 1 March 2013 to 23:00 h on 28 February 2017, with a total of 35,064 data samples, including year, month, day, hour, PM2.5, PM10, SO_2_, NO_2_, CO, O_3_, temperature, barometric pressure, dew point temperature, precipitation, wind speed and wind direction. An example of the data is shown in Fig. 1.

### Data pre-processing

If sample data were missing, the entire sample of missing values was removed and the final processed data of 31,914 was used for the experiments in this article. Next, the character-based data is encoded, with the encoding form referring to Table 1. Taking into account the influence of spatial factors, the PM2.5 concentration in Shunyi was added to the characteristic data as an indicator. The processed data was PCA dimensionality reduction, 97% of the information was retained, and the data feature dimension was 8. Finally, the samples were divided into training and test sets in a ratio of 9:1 and the data were subjected to min-max normalisation to eliminate the effects of order-of-magnitude inconsistencies between features.

**Table 1:** Character data encoding (wind speed).

Wind direction	E	ENE	NNE	N	NNW	NW	NE	SW	SSW	WSW	ESE	SE	S	WNW	SSE	W
Character encoding	1	2	3	4	5	6	7	8	9	10	11	12	13	14	15	16

### Forecasting model and principle

**Figure 2:**
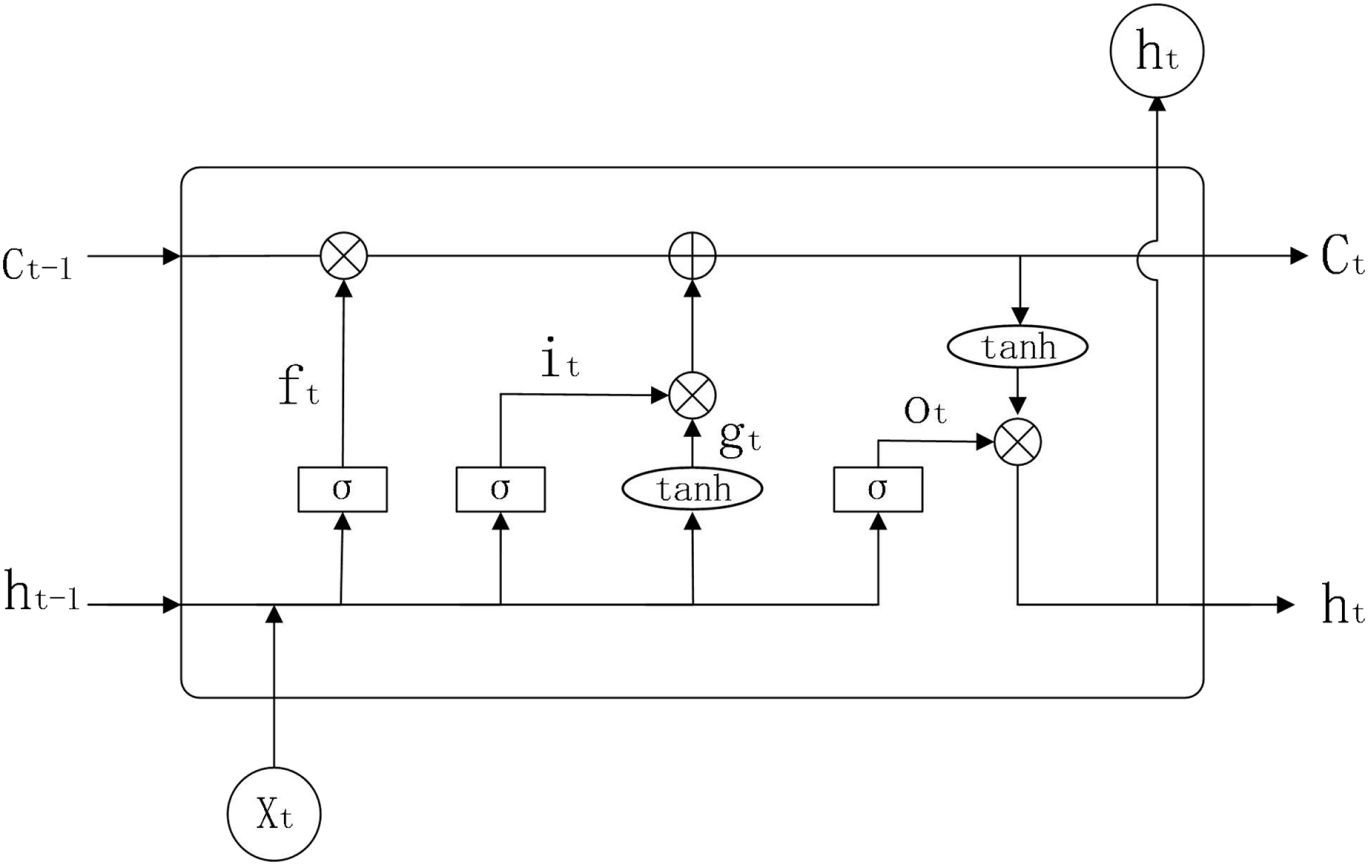
LSTM structure diagram.

The input of a traditional RNN model consists of two parts, namely the current input and the hidden layer information of the previous moment. If the task to be predicted is only relevant to the most recent information, then using an RNN has good results. However, when the task to be predicted also takes into account information from a long time ago, the gradient may disappear or explode when the RNN computes the gradient. In response to this, LSTM introduces three ‘gates’ to control the transmission of information, adding or removing information from neurons. The internal structure of LSTM is shown in Fig 2.

Parameters:

$w_{xf,hf,xi,hi,xg,hg,xo,ho,yh}$: weights for LSTM model in each state

${b_{f,i,g,y}}$: bias value for LSTM models in each state

$\sigma$: sigmoid activation function

${tanh}$: activation function

${f_t}$: forgetting gate

${{i}_t}$: input gate

${{o}_t}$: output gate

${{x}_{t - 1}}$: previous input information

${{x}_t}$: current input information

${{h}_{t - 1}}$: output information of the neuron at the previous moment

${{h}_t}$: output information of the neuron at the present moment

${{C}_{t - 1}}$: neuronal state at the previous moment

${{C}_t}$: neuronal state at the present moment

${{y}_t}$: output results

${{g}_t}$: vector after the ${tanh}$ activation function

${m_t}$: state of neurons after the ${tanh}$ activation function

The working principle of LSTM is shown below.

(1) $${{f}_t} = \sigma ({W_{xf}}{x_t} + {W_{hf}}{x_{t - 1}} + {b_f})$$

(2) $${{i}_t} = \sigma ({W_{xi}}{x_t} + {W_{hi}}{h_{t - 1}} + {b_i})$$

(3) $${{g}_t} = \tanh ({W_{xg}}{x_t} + {W_{hg}}{h_{t - 1}} + {b_g})$$

(4) $${C_{t}} = {C_{{t - }1}}*{{f}_t} + {g_t}*{i_t}$$

(5) $${{o}_t} = \sigma ({W_{xo}}{x_t} + {W_{ho}}{h_{t - 1}} + {b_g})$$

(6) $${{m}_t} = \tanh {C_{\rm t}}$$

(7) $${{h}_t} = {o_t}*{m_t}$$

(8) $${y_t} = {W_{yh}}{h_t} + {b_y}$$

**Figure 3:**
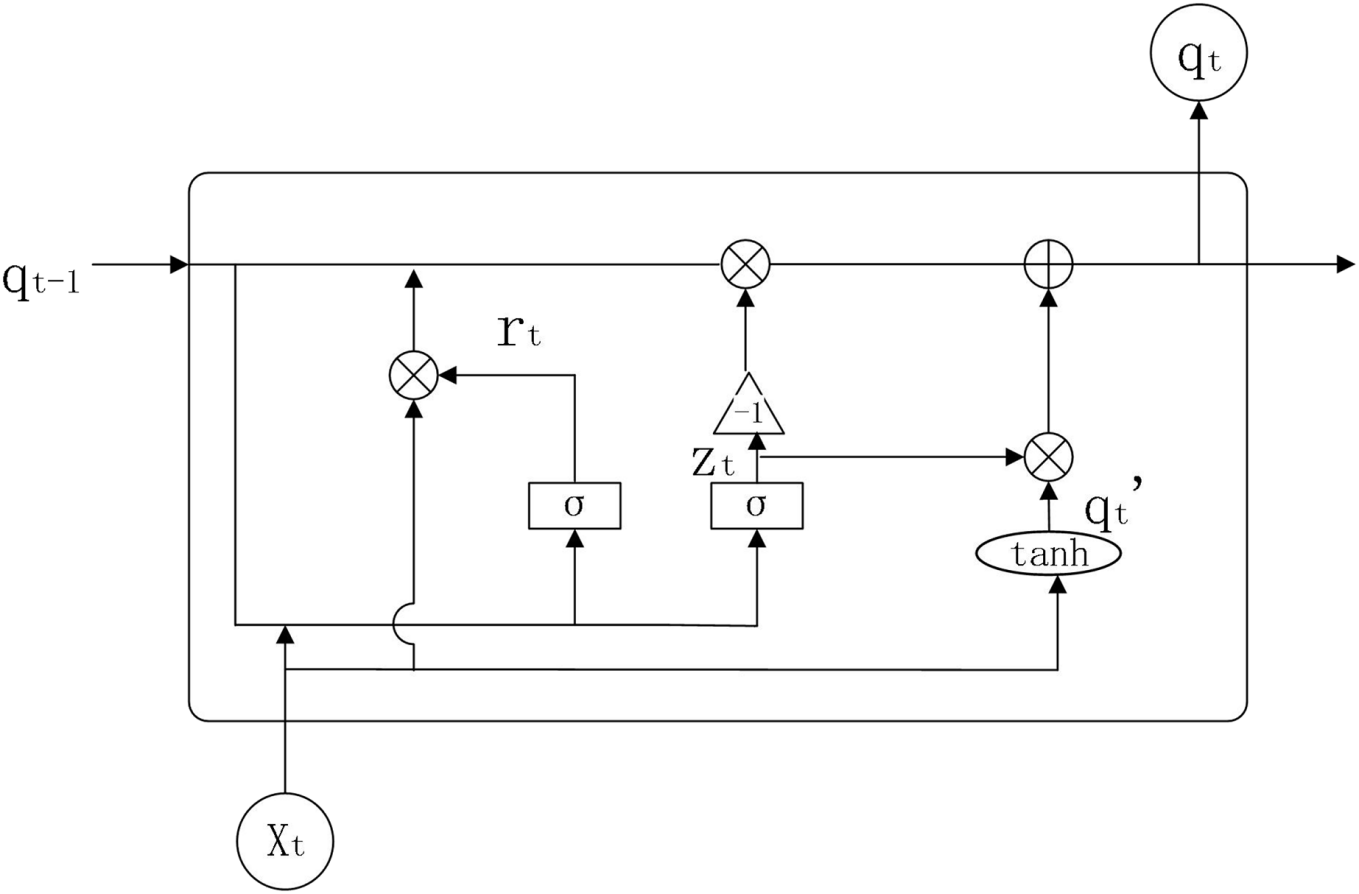
GRU structure diagram.

GRU is an improved version of LSTM, which simplifies the internal structure of LSTM and sets only two gate functions internally to control state information and input information (Li, 2021), that is, reset door and update door. The internal structure of GRU is shown in Fig 3.

Parameters:

${{w}_{xr,hr,xz,hz,p}}$: weights for GRU model in each state

${r_t}$: reset gate

${z_t}$: update gate

${q_{t - 1}}$: output information from neurons at the previous moment

${q_t}$: output information from neurons at the present moment

${{q}_t}^{\prime}$: stores the processed information

The working principle of GRU is shown below.

(9) $${{r}_t} = \sigma ({w_{xr}}{x_t} + {w_{hr}}{q_{t - 1}})$$

(10) $${{z}_t} = \sigma (w{}_{xz}{x_t} + {w_{hz}}{q_{t - 1}})$$

(11) $${q_t}^{\prime} = \tanh ({w_p}{r_t}{q_{t - 1}} + {w_p}{x_t})$$

(12) $${q_t} = (1 - {z_t})*{q_{t - 1}} + {z_t}*{q_t}^{\prime}$$

**Figure 4:**
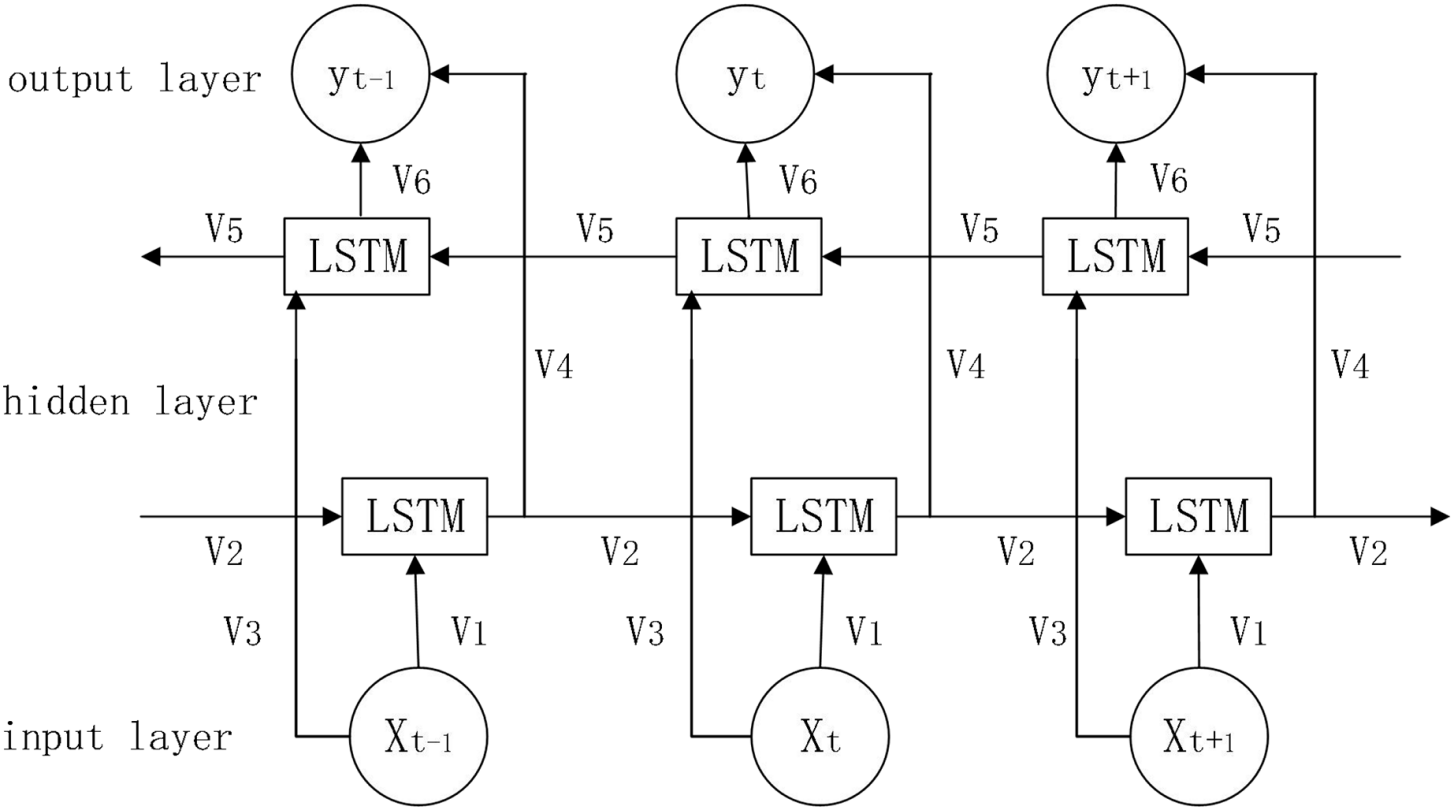
Bi-LSTM network structure diagram.

In prediction, the LSTM algorithm only uses data information from the past moment and does not consider information from the future moment, BI-LSTM can combine information from both front and back directions to make predictions, which will be more accurate to a certain extent (Gao et al., 2020). The network structure diagram is shown in Fig. 4.

Parameters:

${{e}_t}$: forward calculation information

${{e}_t}^{\prime}$: reverse calculation information

${{v}_{\rm i}}$: weights for Bi-LSTM model in each state (i = 1…6)

${{o}_t}$: combination of information from both front and rear directions

The working principle of Bi-LSTM is shown below.

(13) $${e_t} = f({v_1}{x_t} + {{v}_2}{e_{t - 1}})$$

(14) $${e^{\prime}}_t = f({v_3}{x_t} + {{v}_5}{e^{\prime}}_{t + 1})$$

(15) $${o_t} = g({v_4}{e_t} + {v_6}{e^{\prime}}_t)$$

**Figure 5:**
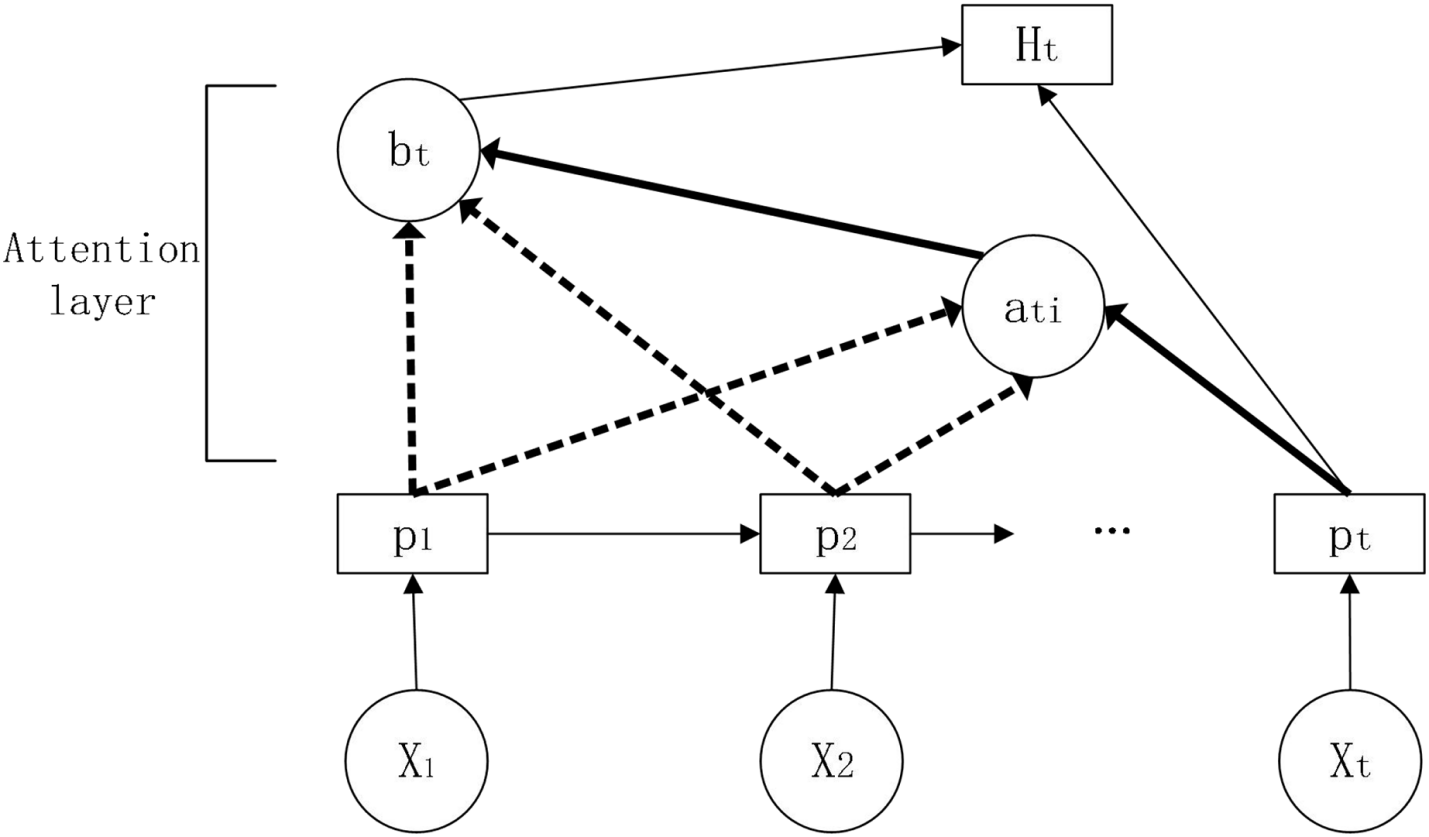
Schematic diagram of the attention mechanism.

The attention mechanism was originally proposed for machine translation and allows the filtering of a small amount of information that is valuable to the predictor from a large amount of information (Chen et al., 2022). The attention mechanism measures the correlation between previous information and the information to be predicted and assigns weights based on the correlation, thus focusing on the information that is more useful for prediction and improving the accuracy of the prediction. Its schematic diagram is shown in Fig. 5.

In this article, we introduce the attention mechanism in the LSTM model and construct the LSTM-Attention, a long and short term memory network model based on the attention mechanism.

Parameters:

${w}$, ${{w}_{{c,y}}}$: weights for LSTM-Attention model in each state

${\alpha _{{ti}}}$: attention weights

${{p}_{t}}$: state of the hidden layer at this moment

${{p}_{i}}$: state of the hidden layer for the input information at the corresponding moment

${{s}_{{ti}}}$: weighted score

${{H}_{t}}$: final hidden layer state of the current output

${l}$: bias value

First, the information is input to the LSTM to obtain the hidden layer state, then the attention score is calculated and the similarity between the past and current information is evaluated based on the obtained hidden layer information. Adding a fully connected neural network layer to train the parameter matrix (Wang et al., 2021) and obtain the weight score by dot product operation.

(16) $${{\rm s}_{ti}} = {p_t}^Tw{p_i}$$

Secondly, the obtained attention score is normalized by the function to obtain the attention weight.

(17) $${\alpha _{{ti}}} = {softmax}({s_{ti}}) = \displaystyle{{\exp ({s_{ti}})} \over {\sum {\exp ({s_{ti}})} }}$$

Next, the attention weights obtained above are weighted and summed with the previous hidden state values.

(18) $${{b}_{t}} = \sum\limits_i^n {{\alpha _{{ti}}}{p_i}}$$

Finally, the obtained weighted summation result is spliced with the current hidden state. After the fully connected layer, the final hidden state is obtained through the ${tanh}$ activation function.

(19) $${H_{\rm t}} = \tanh ({w_c}[{b_t};{p_t}])$$

Through the fully connected layer, the output result $Y$ is obtained.

(20) $$Y = {{\rm w}_y}{H_t} + l$$

LightGBM is a distributed gradient boosting framework based on the decision tree algorithm. This article has a large amount of data and runs inefficiently, while the light model can improve the computing efficiency. In this article, the lightGBM model and the above LSTM-Attention model are introduced to construct the integrated model lightGBM+LSTM-Attention by the error reciprocal method. Firstly, the prediction results are obtained by lightGBM model and LSTM-attention model respectively. Then, the two models are integrated by the error reciprocal method and the weights are redistributed. The prediction model with small error can obtain large weight value after calculation, while the prediction model with large error will obtain relatively small weight value. Thus, the overall prediction error can be reduced.

Parameters:

${er}{{r}_{lig}}$: error value of lightGBM model

${er}{{r}_{lstm - att}}$: error value of LSTM-attention model

${{w}_{lig}}$: weight value of lightGBM model

${{w}_{lstm - att}}$: weight value of LSTM-attention model

${{y}_{1{i}}}$: predicted value of lightGBM model (i = 1..n)

${{y}_{2i}}$: predicted value of LSTM-attention model (i = 1..n)

${{y}_{now}}$: predicted value of integrated model

First, the weights are obtained from the two model prediction values.

(21) $${{w}_{lig}} = \displaystyle{{er{r_{lstm - att}}} \over {er{r_{lstm - att}} + er{r_{lig}}}}$$

(22) $${{w}_{lstm - att}} = \displaystyle{{er{r_{lig}}} \over {er{r_{lig}} + er{r_{lstm - att}}}}$$

Then, weights are assigned to the predicted values to increase the weight of the predicted accurate values.

(23) $${{y}_{now}} = {w_{lig}}{y_{1i}} + {w_{lstm - att}}{y_{2i}}$$

## Results

According to the data used, the data are constructed according to the time series, and the samples are divided into training samples and test samples. The model is constructed to predict air quality and focuses on comparing the prediction accuracy of different improved models of LSTM with the model proposed in this article. Based on the prediction results of the model, the fit and error of the model were compared to obtain the optimal model for this experiment.

The LSTM related models constructed in this article have two layers. The number of neurons in each layer is set to 128, 64, and one dropout layers is added to prevent the occurrence of overfitting. The final output is realized by a fully connected layer. The loss function of the model is MAE and the optimizer is Adam. The learning rate is 0.0005.

The data are brought into the 12 models constructed in this article for modeling. This article uses coefficient of Determination (R2), mean absolute error (MAE), and root mean square error (RMSE) to measure prediction accuracy. When the value of R2 is closer to 1 or the RMSE and MAE are smaller, the prediction is more accurate. The results show that the lightGBM model has the highest prediction accuracy among the machine learning algorithms, and the prediction accuracy of deep learning methods is generally higher than that of machine learning methods. Among them, the integrated model constructed in this article has the highest accuracy, reaching 0.969, which significantly improves the prediction accuracy of the single LSTM model and its improved model.

## Discussion

**Figure 6:**
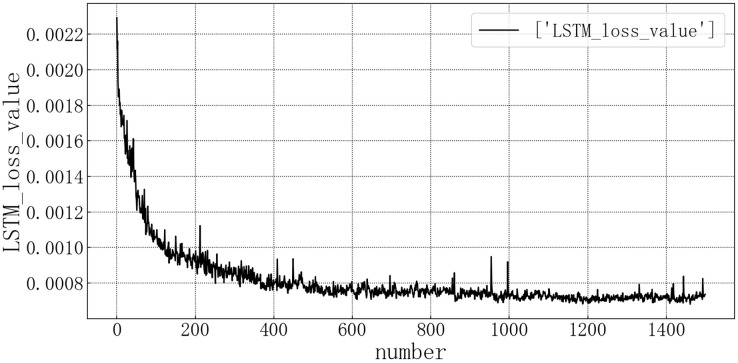
Loss diagram of LSTM test set.

Bring the data into the constructed LSTM model for modeling. After the model is trained, the test set is brought into the test set to evaluate the prediction situation. The test set loss rate is shown in the Fig. 6. The horizontal axis represents the number of model iterations, and the vertical axis represents the loss rate. The model loss rate converged below 0.0008 and tended to be stable, reaching the optimal level in this article.

**Figure 7:**
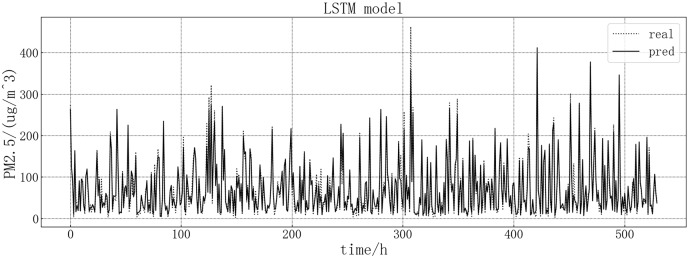
LSTM model fitting diagram.

The fitting diagram between the predicted value and the real value of the LSTM model is shown in Fig. 7. According to the fitting of the prediction results of the LSTM model with the real results, it can be seen that the LSTM model in this article has a good prediction effect on PM2.5, but there are still some inaccurate predictions, such as the decline point of the value in the chart, which may be contrary to the development trend of the true value.

**Figure 8:**
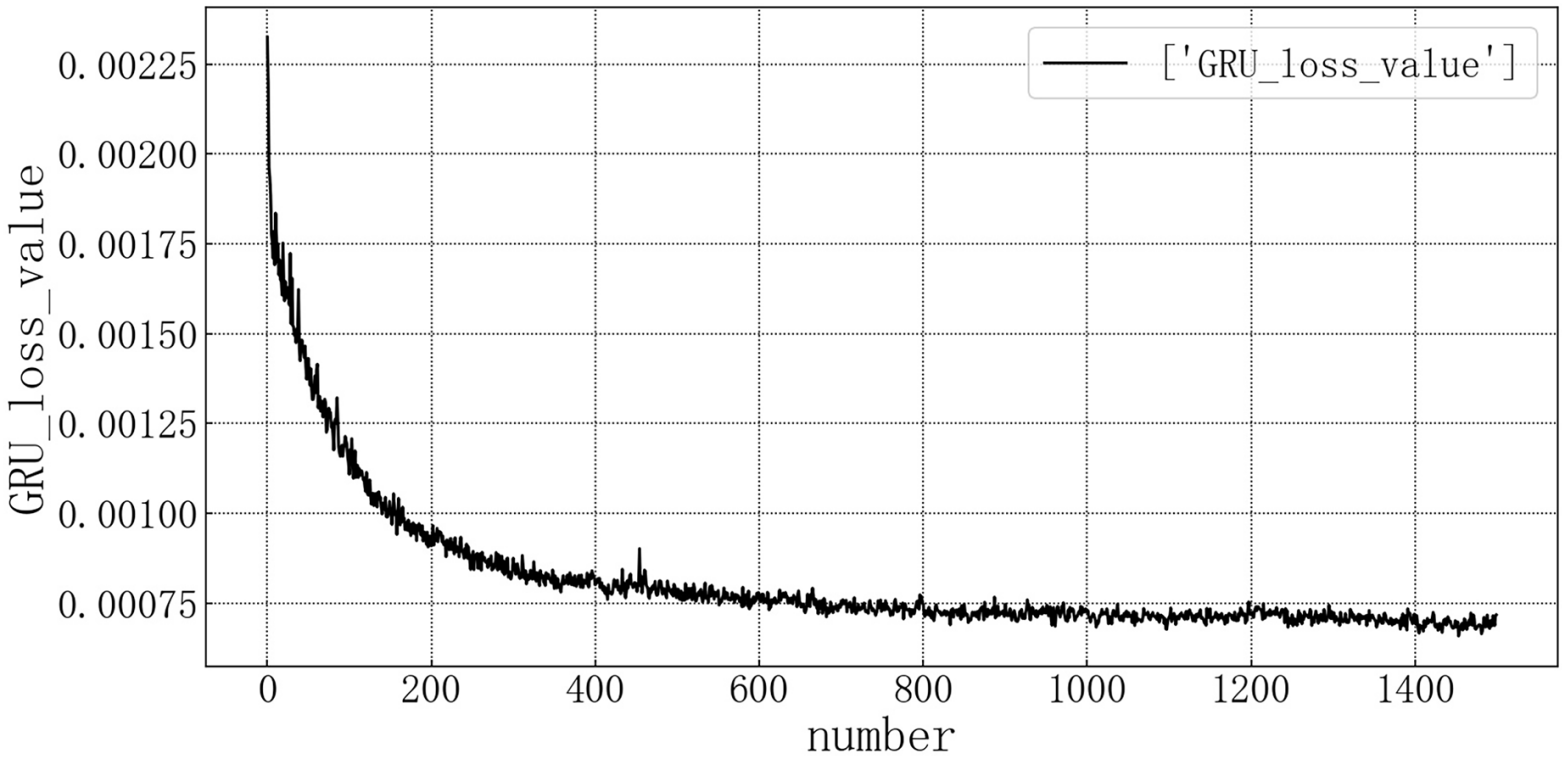
GRU test set loss diagram.

Bring the data into the constructed GRU model for modeling. It is verified on the test set, and the test set loss rate is shown in Fig. 8. The model loss rate converged below 0.00075 and tended to be stable, reaching the optimal level in this article.

**Figure 9:**
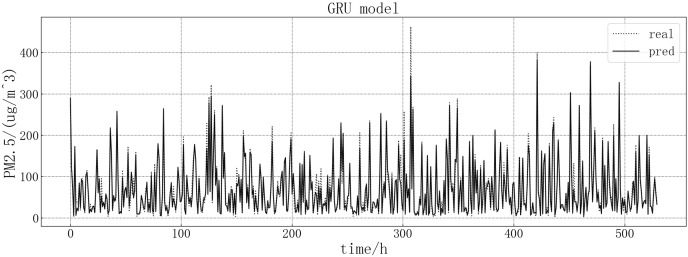
GRU model fitting diagram.

The fitting between the predicted value and the real value of GRU model is shown in Fig. 9. According to the fitting of the predicted results of the GRU model with the real results, it can be seen that the prediction effect of the GRU model on the PM2.5 of this article is better than LSTM model. It is more accurate when predicting stationary values, and predicting a trend consistent with the true value at the point of dip in the value, but there are large value inaccuracies.

**Figure 10:**
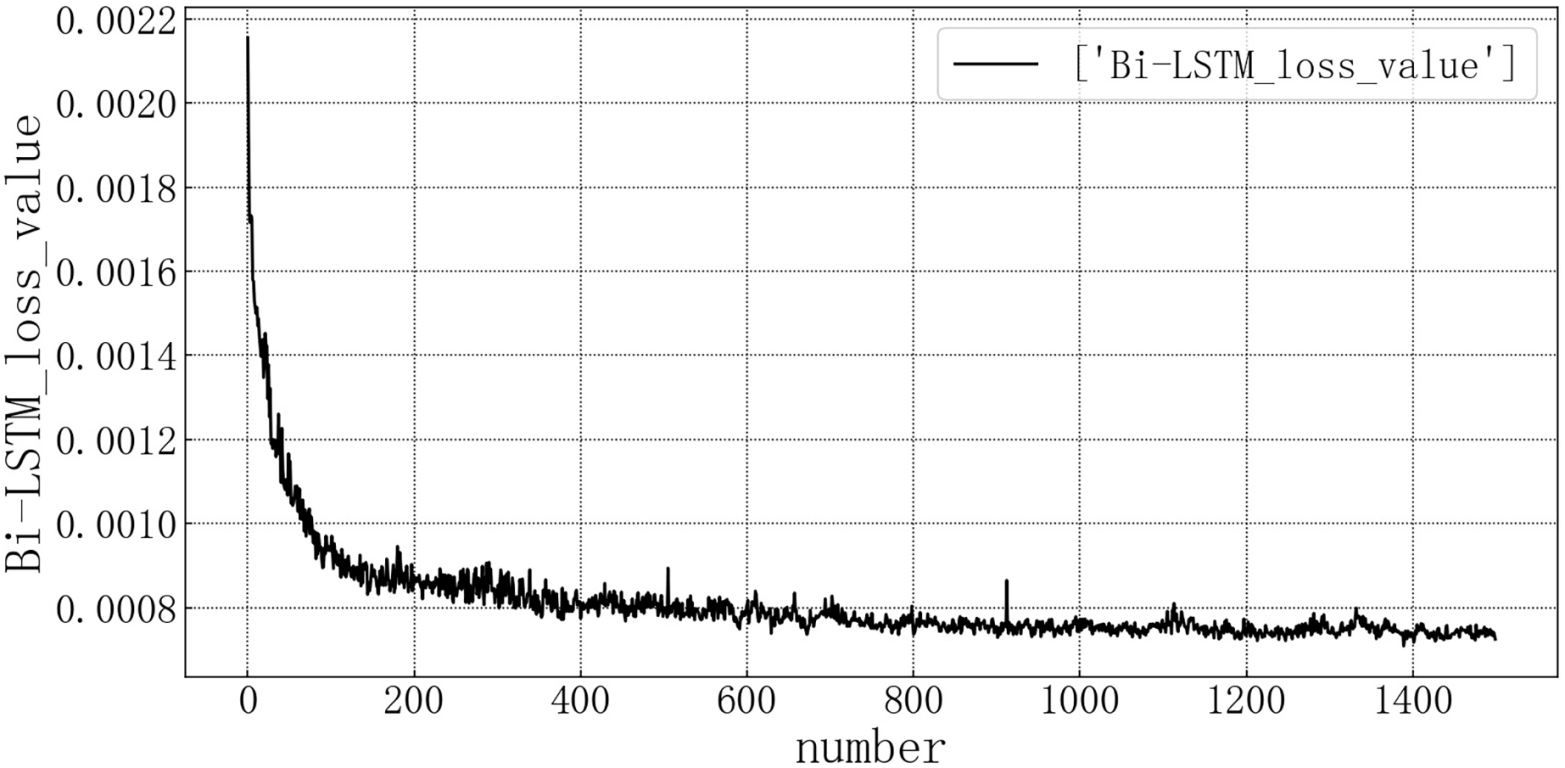
Bi-LSTM test set loss diagram.

Bring the data into the constructed Bi-LSTM model for modeling. After training the model, use the test set to verify the prediction. The loss rate of the test set is shown in Fig. 10. The model loss rate converged below 0.0008 and tended to be stable, reaching the optimal level in this article.

**Figure 11:**
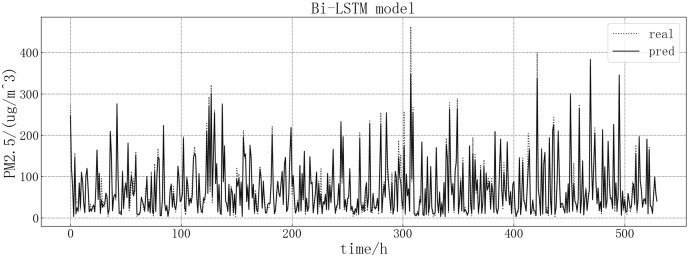
Bi-LSTM model fitting diagram.

The fitting of the predicted value and the actual value is shown in Fig. 11. According to the fitting of the prediction results of the Bi-LSTM model with the real results, it can be seen that the prediction accuracy of the Bi-LSTM model is higher than that of the LSTM model and is comparable to that of the GRU model. At some sudden changes in the falling value, the forecast works very well, but the error is large in the prediction of larger values.

**Figure 12:**
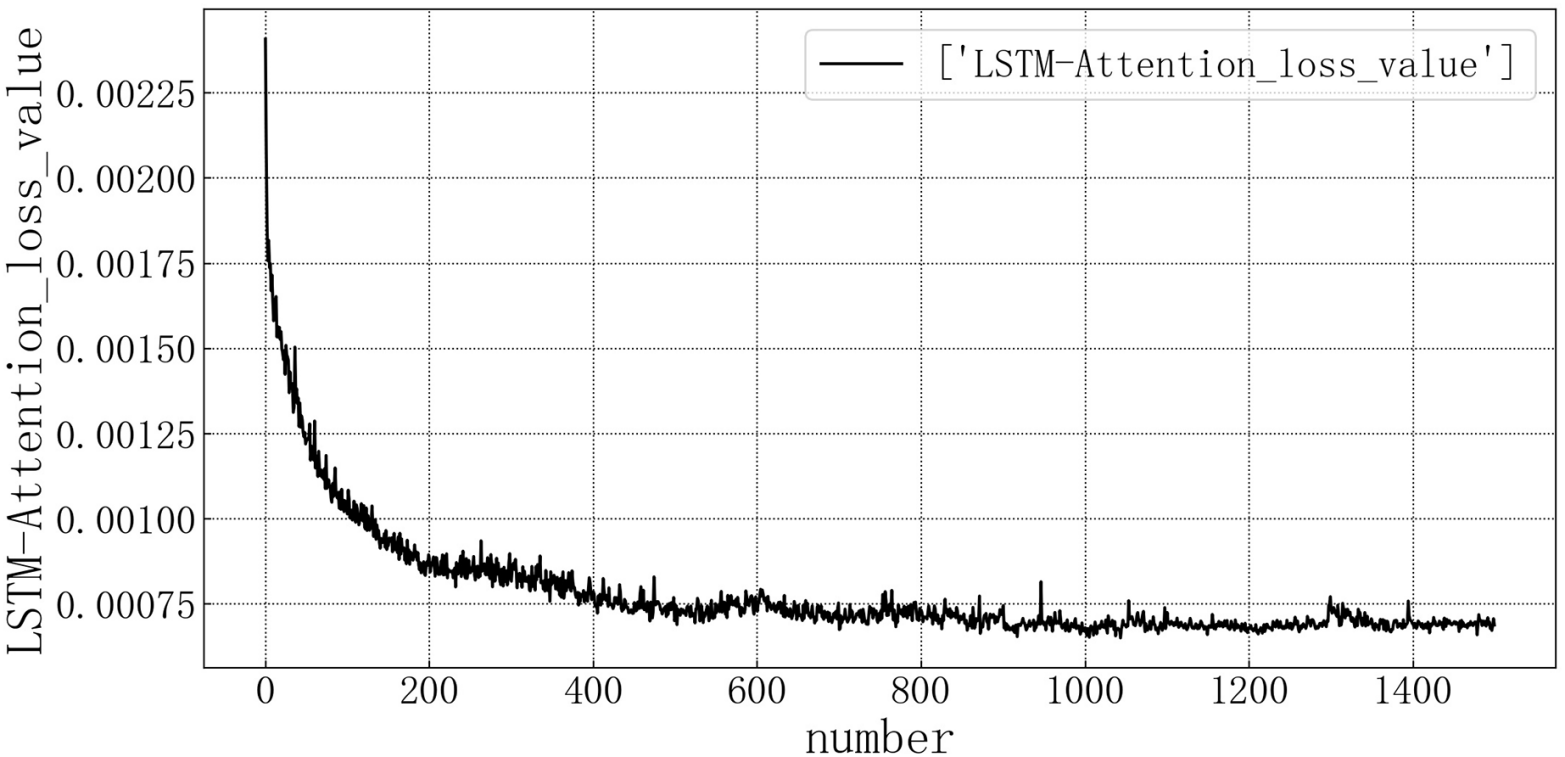
LSTM-Attention test set loss diagram.

Bring the data into the constructed LSTM-Attention model for modeling. The test set loss rate is shown in Fig. 12. The model loss rate converged below 0.00075 and tended to be stable, reaching the optimal level in this article.

**Figure 13:**
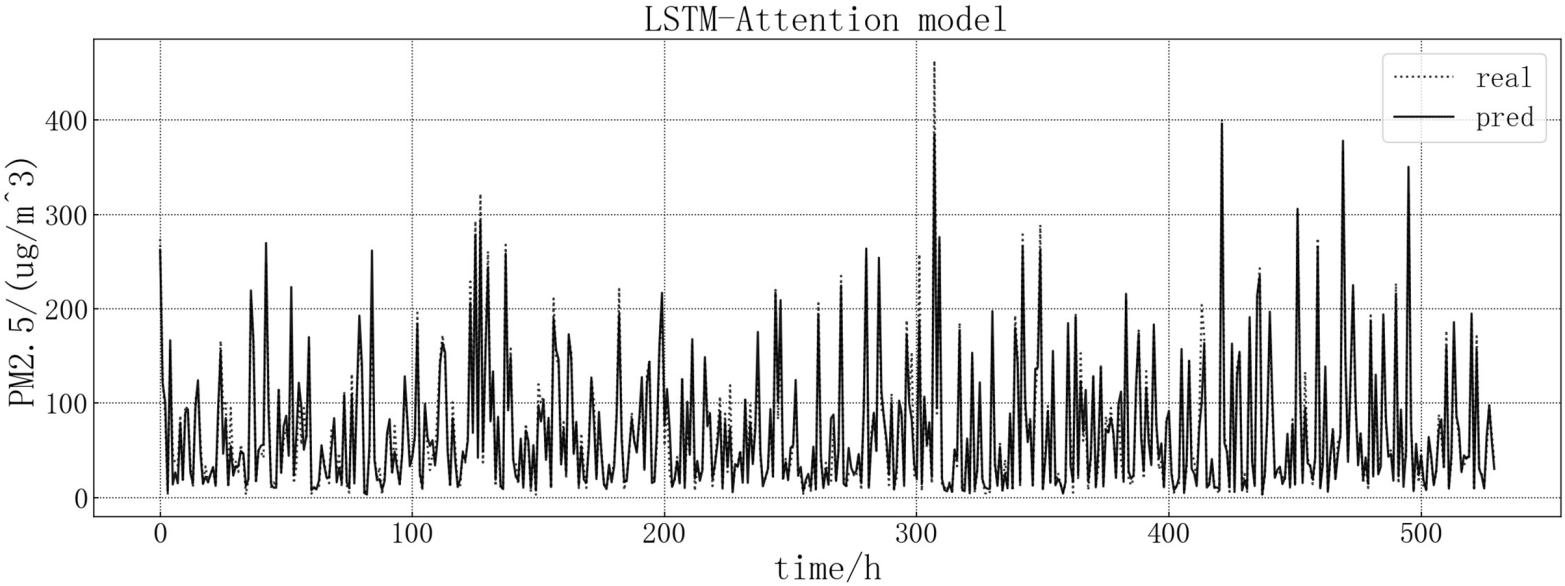
LSTM-Attention model fitting diagram.

Bring the data into the constructed LSTM-Attention model for modeling. The fitting of the predicted value and the actual value is shown in Fig. 13. According to the fitting of the prediction results of the LSTM-Attention model with the real results, it can be seen that the prediction accuracy of the LSTM-Attention model is higher than that of LSTM correlation models and have improved prediction accuracy compared to other models on some larger values.

**Figure 14:**
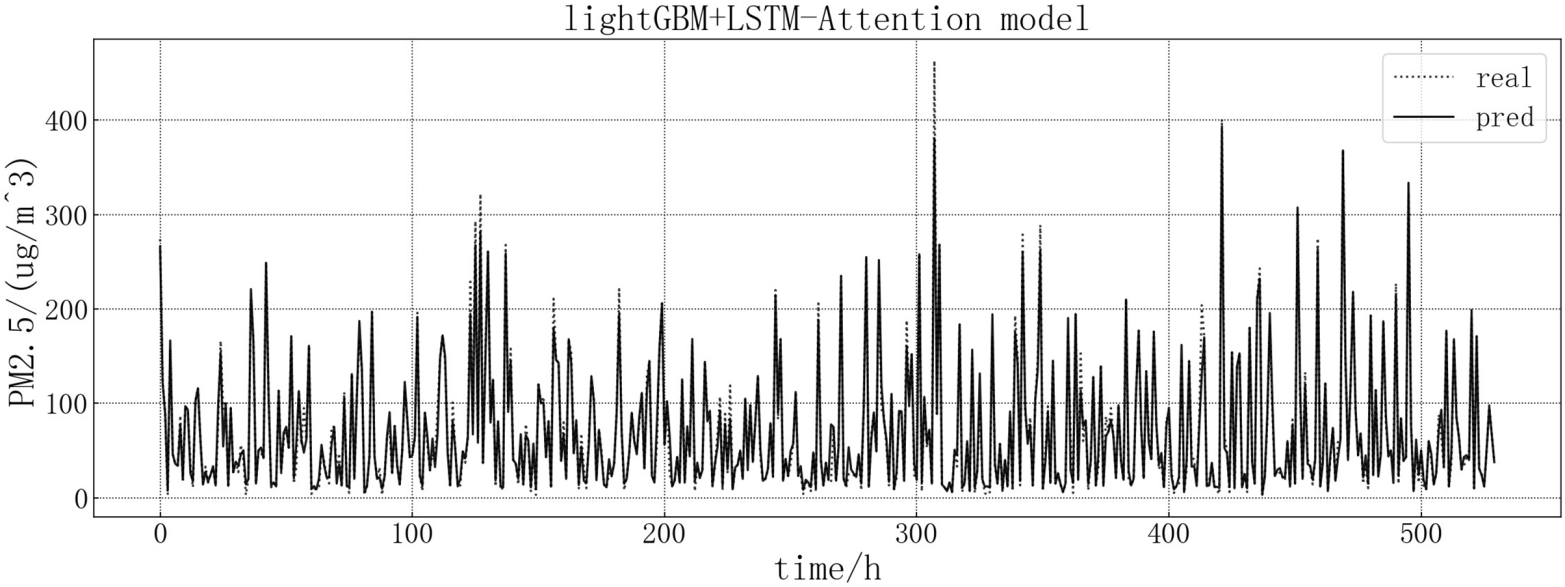
lightGBM+LSTM-Attention model fitting diagram.

Bring the data into the constructed lightGBM-LSTM-Attention model for modeling. The fitting of the predicted value and the actual value is shown in Fig. 14. Based on the fit of the ensemble model results with the actual results, we can see that the prediction accuracy of the ensemble model is greatly improved, and the prediction is more accurate than other models at some large values or dip points.

**Figure 15:**
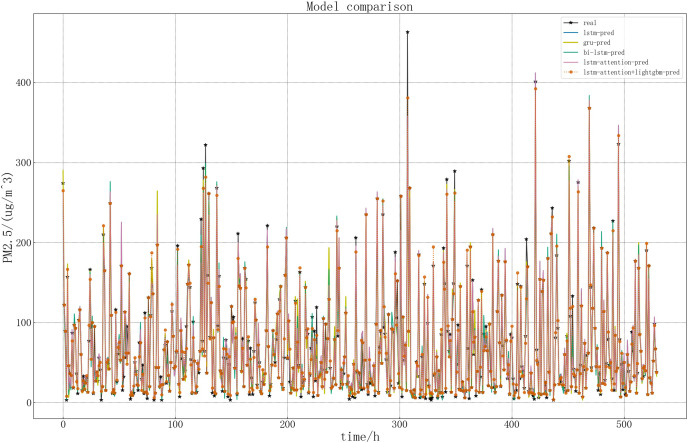
Comparison of five models.

In order to more intuitively show the performance of the LSTM related models in this article, draw a picture of the predicted and real values of LSTM related models. The specific comparison is shown in Fig. 15.

According to the above figure and Table 2, it can be seen intuitively that in the five models, the predicted result curve basically coincides with the real result, that is, the predicted general trend is consistent with the real value. Compared with the other four models, the accuracy of LSTM model in predicting PM2.5 concentration is lower. The lightGBM+LSTM-Attention model performs better in this article, which is closer to the real value. This is because the improved model is more accurate in capturing important information and can retain and utilize the previous information. At the same time, the error reciprocal method can reduce the weight of the imprecise prediction value and increase the weight of the accurate prediction value, so that the predicted value can be modified on the whole and better prediction effect can be achieved.

**Table 2:** PM2.5 error index predicted by different models.

Model indicators	R^2^	RMSE	MAE
Random forests	0.878	25.18	15.73
AdaBoost	0.877	25.30	16.00
GBRT	0.867	26.03	16.35
XGBoost	0.876	25.39	16.01
LightGBM	0.886	24.36	15.29
SVR	0.821	30.55	21.10
Lasso	0.854	27.58	17.31
LSTM	0.939	17.89	9.95
Bi-LSTM	0.940	17.74	9.60
GRU	0.940	17.65	9.45
LSTM-Attention	0.946	16.80	9.04
lightGBM+LSTM-Attention	0.969	12.80	5.25

## Conclusions

In this article, 12 models were built to predict the concentration of PM2.5 in the air. These are random forest, AdaBoost, GBRT, XGBoost, lightGBM, SVR, Lasso, LSTM, GRU, Bi-LSTM, LSTM-Attention, lightGBM+LSTM-Attention. After comparing the performance of the different models on the same test set, it can be concluded that for the predictions in this article, the fitting accuracy of both the LSTM and the improved model exceeds 0.9. Prediction accuracy is generally higher than traditional machine learning models. The lightGBM+LSTM-Attention model showed the best prediction accuracy, with RMSE improvements of 5.09, 4.94, 4.85 and 4.0 compared to the other LSTM models.

Therefore, the lightGBM+LSTM-Attention model established in this article can achieve a more accurate prediction of PM2.5 concentration, which provides a new way of thinking for air quality prediction and is of great value for future air quality evaluation. In terms of space, this article only considers the possible impact of nearby regions on it. In the future, multiple regions could be considered in order to improve the progress of the predictions.

## Supplemental Information

10.7717/peerj-cs.1187/supp-1 Supplemental Information 1 Air quality prediction code.

10.7717/peerj-cs.1187/supp-2 Supplemental Information 2 Air quality prediction dataset.

## Additional Information and Declarations

### Data Availability

The following information was supplied regarding data availability: The raw data and the code are available in the Supplemental Files.
