# Supplementary figures and images for: Research on air quality prediction based on improved long short-term memory network algorithm
Source: PeerJ Comput Sci. 2022 Dec 20;8:e1187. doi: 10.7717/peerj-cs.1187 (PMC10280268; doi:10.7717/peerj-cs.1187)

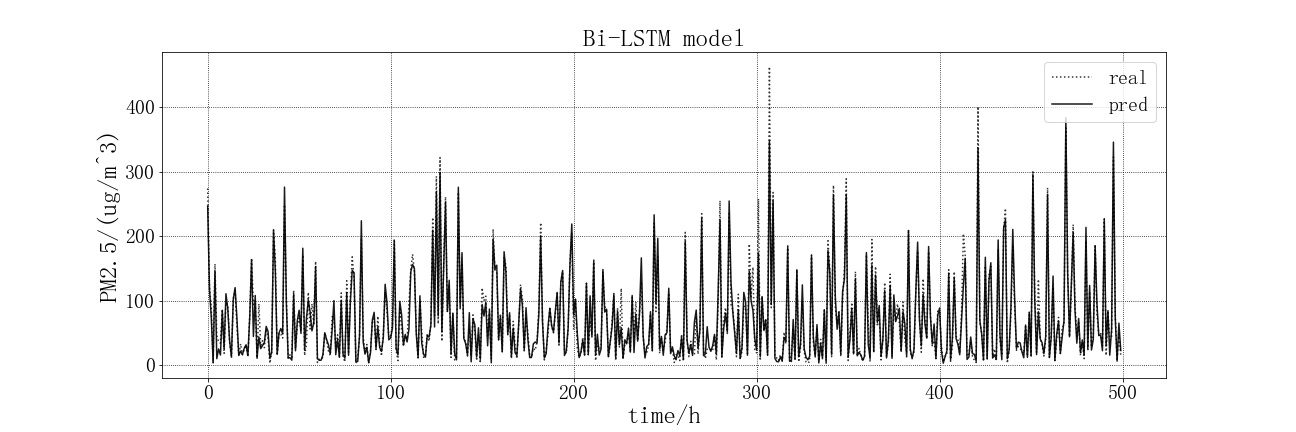

Supplement: Supplemental Information 1 [file peerj-cs-08-1187-s001.zip › code/Bi-LSTM─Γ║╧═╝500.jpg]

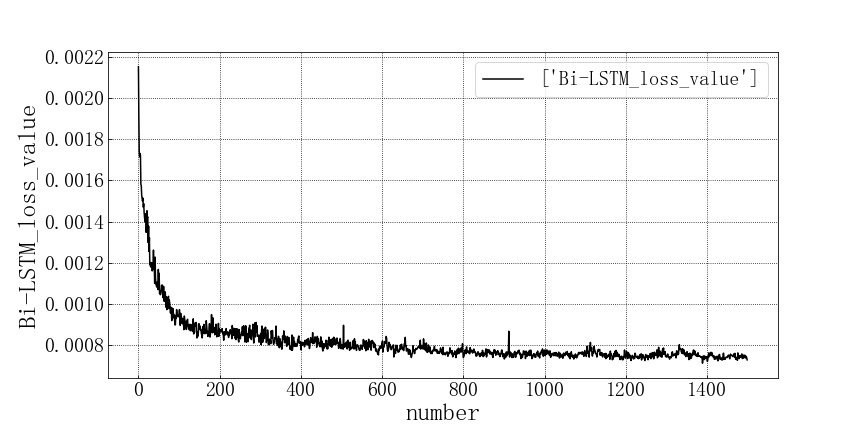

Supplement: Supplemental Information 1 [file peerj-cs-08-1187-s001.zip › code/Bi-LSTM╦≡╩o═╝.jpg]

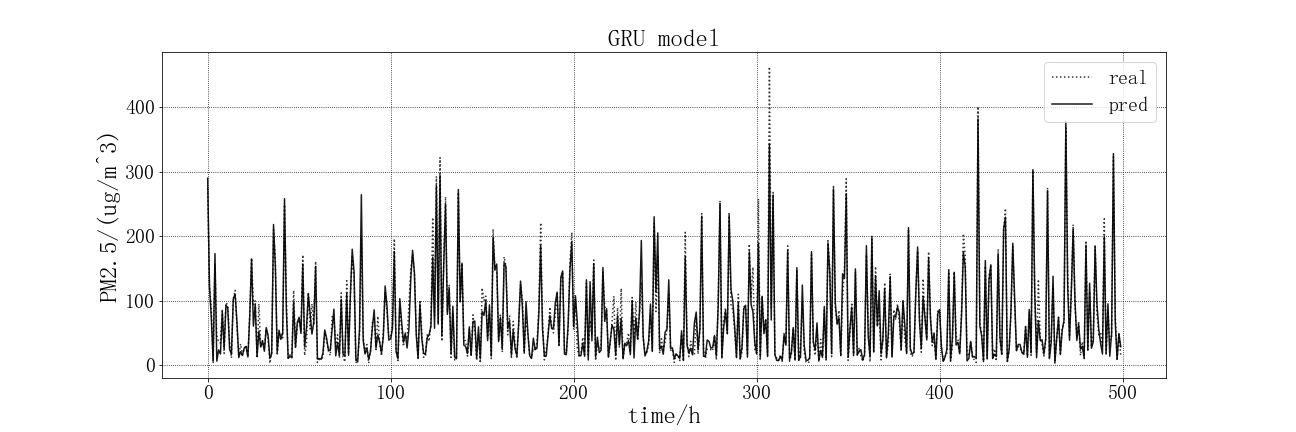

Supplement: Supplemental Information 1 [file peerj-cs-08-1187-s001.zip › code/GRU─Γ║╧═╝500.jpg]

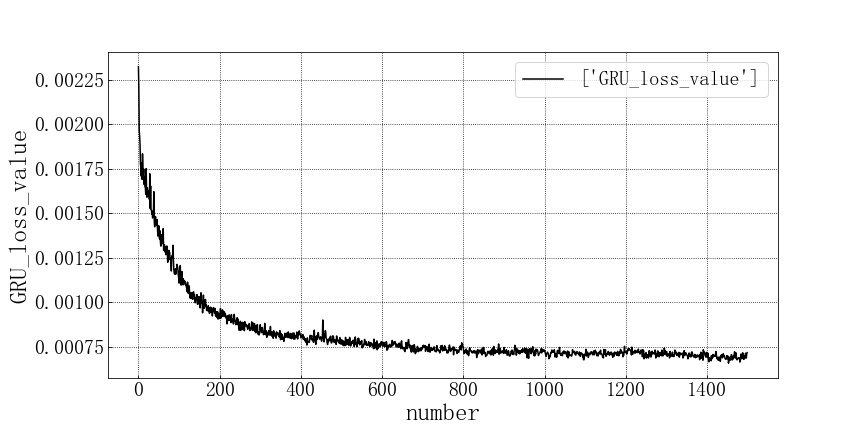

Supplement: Supplemental Information 1 [file peerj-cs-08-1187-s001.zip › code/GRU╦≡╩o═╝.jpg]

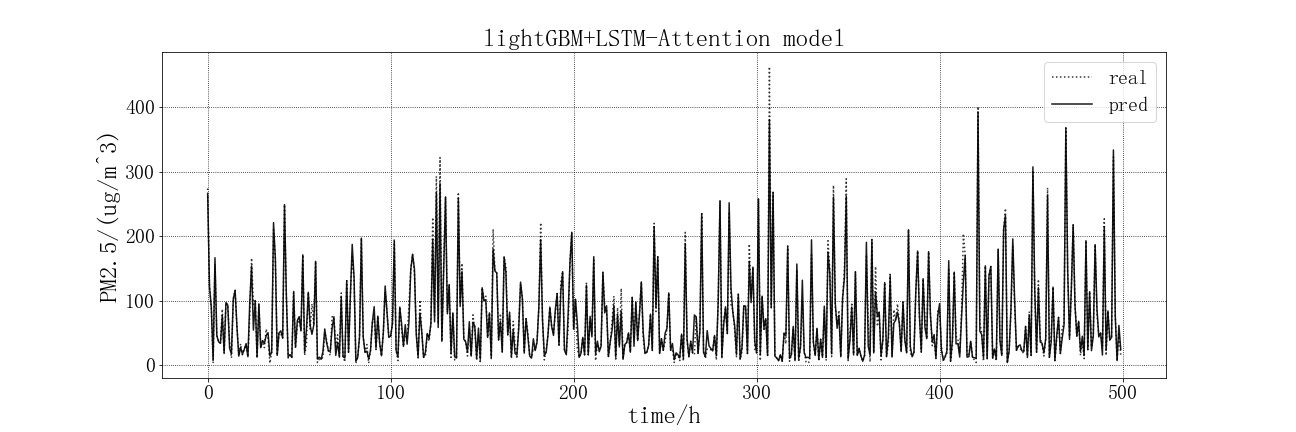

Supplement: Supplemental Information 1 [file peerj-cs-08-1187-s001.zip › code/lightGBM+LSTM-Attention─Γ║╧═╝500.jpg]

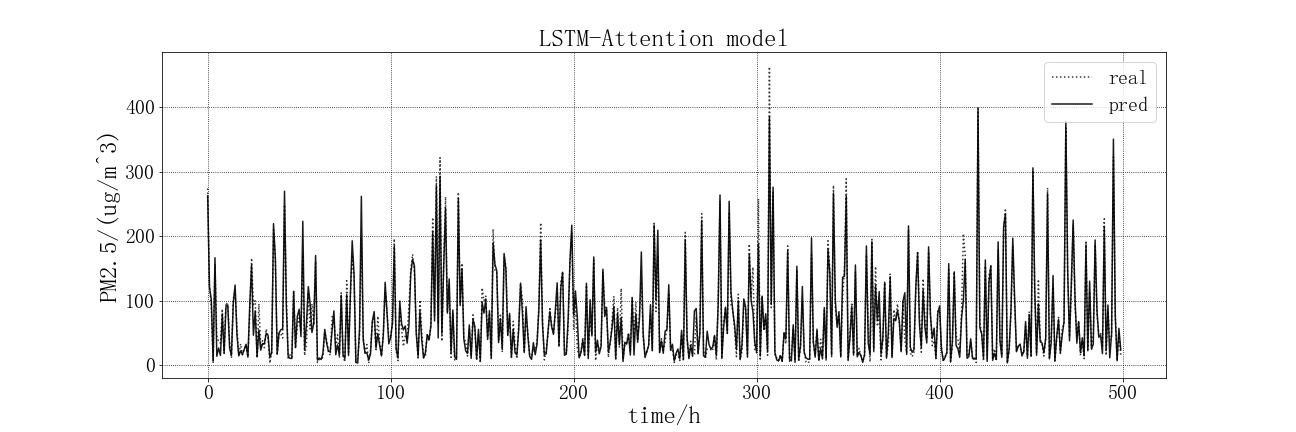

Supplement: Supplemental Information 1 [file peerj-cs-08-1187-s001.zip › code/LSTM-Attention─Γ║╧═╝500.jpg]

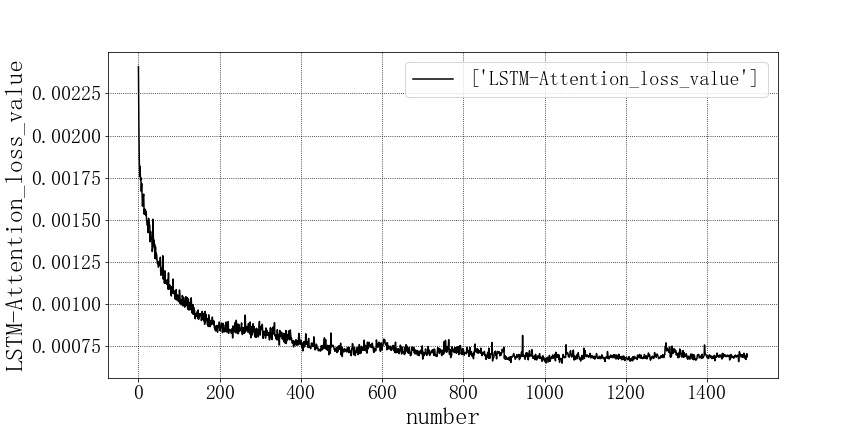

Supplement: Supplemental Information 1 [file peerj-cs-08-1187-s001.zip › code/LSTM-Attention╦≡╩o═╝.jpg]

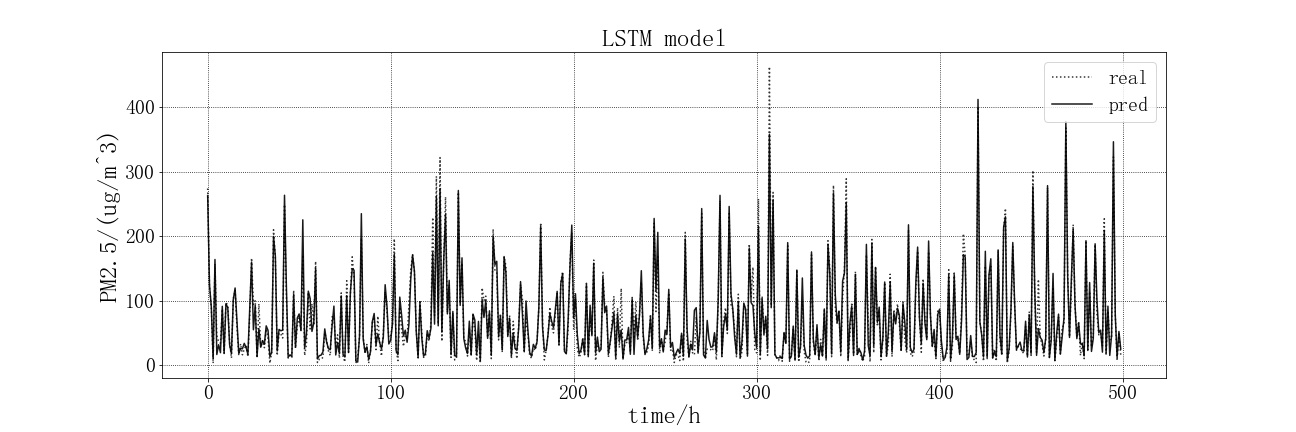

Supplement: Supplemental Information 1 [file peerj-cs-08-1187-s001.zip › code/LSTM─Γ║╧═╝500.jpg]

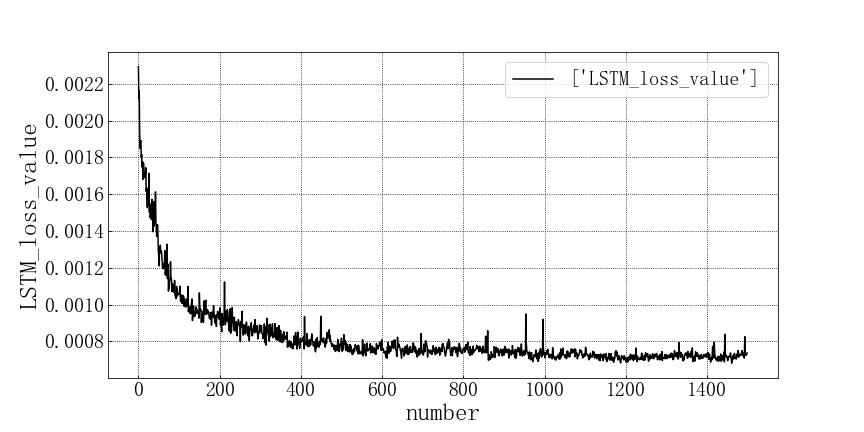

Supplement: Supplemental Information 1 [file peerj-cs-08-1187-s001.zip › code/LSTM╦≡╩o═╝.jpg]

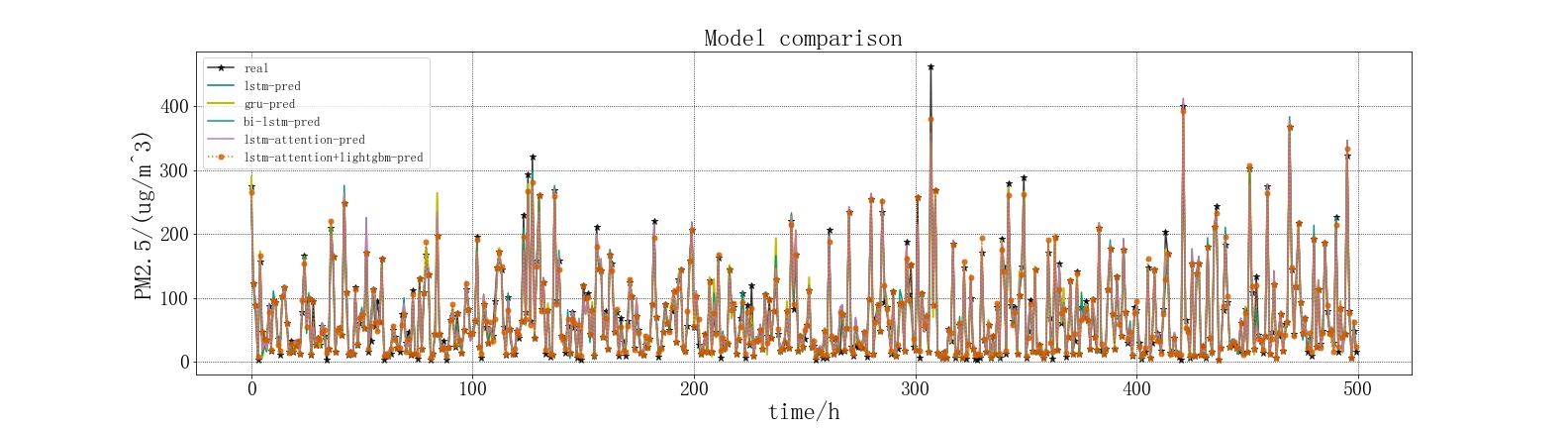

Supplement: Supplemental Information 1 [file peerj-cs-08-1187-s001.zip › code/╫▄╠σ─Γ║╧═╝500.jpg]
